# Policy options to transform food systems for affordable and healthy diets in South Africa: a systematic review with stakeholder prioritization

**DOI:** 10.3389/fnut.2026.1855954

**Published:** 2026-09-04

**Authors:** Wegayehu Fitawek, Lukhangele Mgweba, Olwethu Loki

**Affiliations:** Department of Agricultural Economics, Extension and Rural Development, University of Pretoria, Pretoria, South Africa

**Keywords:** food policy, food systems, healthy diets, policy options, transform

## Abstract

**Introduction:**

South Africa’s food system is struggling to deliver nutritious, affordable diets, leaving many people unable to access healthy food. Furthermore, the country lacks a comprehensive food system policy, relying instead on policies that address specific components of food security. This study investigated cost-effective, feasible, and adaptable policy options to transform South Africa’s food system towards affordable, healthy diets.

**Methods:**

A systematic review was conducted in accordance with the Preferred Reporting Items for Systematic Reviews and Meta-Analyses (PRISMA) guidelines. Scopus, PubMed, Web of Science, and other sources were searched for English-language publications from 2013 to 2023. Of the 340 records identified, 20 food systems transformation reports met the eligibility criteria, with 70% focusing on global contexts and 30% on Africa. The identified prioritised policy options were subsequently validated through an online survey of food system stakeholders.

**Results:**

Agricultural policy options were prioritised most frequently (25%), followed by supply chain and governance interventions (20% each), regulatory measures (15%), and education and awareness interventions (10%). Financial, research, technology, and innovation policy options were least prioritised (5% each). Overall, 20 prioritised policy options were identified. The findings indicate that no single policy option is sufficient to improve the affordability of nutritious diets in South Africa. Instead, a coordinated package of complementary interventions across agriculture, governance, supply chains, regulation, finance, education, and research and innovation is required.

**Conclusion:**

These prioritised policy options provide policymakers with an evidence-informed framework for identifying and prioritising interventions that can support healthier diets and contribute to the transformation of South Africa’s food systems.

## 1 Introduction

Over the past five decades, global food systems have shifted from predominantly rural structures to more industrialised and consolidated models, influencing people’s diets, nutrition, livelihoods, and overall health (1). Additionally, food systems have been evolving in ways that undermine the affordability of healthy diets. Cheap, ultra-processed foods have become widely available, while fresh, nutritious foods are less affordable and less accessible to many, especially those experiencing poverty in low- and middle-income countries (2). Food systems in Africa and globally are increasingly affected by economic shocks, pandemics, climate change, and related crises that disrupt food production, distribution, and dietary outcomes (3).

According to the High-Level Panel of Experts (4), a food system is defined as all the elements (people, environment, inputs, processes, infrastructure, institutions) and activities that relate to the production, processing, distribution, preparation, and consumption of food, as well as the consequences of these activities. Enhancing the affordability of healthy diets will require transforming food systems through policies tailored to individual countries, taking into account different trade-offs and leverage points (5). In 2024, approximately 673 million people (8.2%) of the global population and around 307 million people (46%) in Africa faced hunger (6). Looking ahead, it is estimated that by 2030, about 512 million people worldwide may be affected by hunger, with roughly 60% of them in Africa (6). This underscores the urgent need to strengthen the sustainability of food systems across Africa to ensure universal access to healthy, sustainable diets (7). The need to transform food systems has also been identified in the Global Sustainable Development Report 2019 as a means to accelerate progress toward the 2030 Agenda for Sustainable Development (8). The Kampala Declaration, adopted by the African Union in January 2025, is a decade-long strategy and action plan for transforming Africa’s agrifood systems (9).

The transformation of food systems has become a central priority in addressing the growing burden of malnutrition in all its forms. According to (10), policymakers can implement a wide range of measures to enable and incentivise food systems to provide healthy diets while addressing wasting, stunting, underweight, obesity, and diet-related non-communicable diseases. This shift has contributed to the emergence of the food systems approach, which responds to the increasing complexity and evolving nature of food policy challenges. The approach recognises that achieving food and nutrition security requires a comprehensive understanding of the interconnected processes and actors involved throughout the food system, from production and processing to distribution, consumption, and waste management (11).

The food systems approach examines the multiple and interacting causes of food and nutrition challenges while considering the social, environmental, and economic consequences of potential interventions. Its primary objective is to promote transformative, system-wide change rather than addressing isolated components of the food system. Unlike conventional approaches, which often focus on individual sectors such as agricultural production, nutrition-specific interventions, commodity policies, food safety, or trade and are typically implemented within the mandate of a single ministry, the food systems approach adopts a holistic and integrated perspective. It acknowledges the interdependencies among agriculture, health, environmental sustainability, markets, and livelihoods, and seeks to overcome the limitations of fragmented, sector-specific strategies. Furthermore, it encourages collaboration among diverse stakeholders and the coordination of policies across sectors and levels of governance, thereby fostering more coherent and effective solutions to complex food system challenges.

The South African food system is highly complex and is characterised by a paradox, in which the country produces sufficient food, many households remain food insecure, and is shaped by inequalities rooted in colonialism and apartheid policies (12). Despite this, the country has a positive national food balance and well-developed food security and nutrition policies; the food systems simultaneously generate undernutrition and overnutrition, rely on unsustainable production practices, and show slow progress toward transformation. Consequently, large commercial farms continue to operate alongside a vast number of smallholder farmers (13). Over 7,000 commercial farmers account for over 80 percent of agricultural production, while over 2 million smallholder farmers account for the remaining (14). South Africa’s increasingly industrialised and concentrated food systems make it even more difficult to address food-related health risks. Nearly half of the adults in South Africa are overweight or obese because most of the foods are highly processed, nutritionally poor, energy-dense foods that are high in saturated fats, sodium, added sugars, synthetic additives, and preservatives, are readily available, affordable, and socially acceptable (15, 16).

There is no evidence of a nationally representative, regularly updated, healthy and balanced food basket in South Africa. Existing food baskets do not adequately reflect either the typical diets consumed by the population or the minimum nutritional needs of households and individuals. Food baskets are defined sets of food items used to estimate the cost of achieving specific objectives, such as representing a population’s typical dietary patterns or meeting minimum nutritional requirements. They are widely used to monitor food prices, assess the affordability of healthy diets, and inform food security and social policy. Consequently, there is a need to develop a nationally representative healthy food basket to improve understanding of the cost and affordability of basic healthy foods. Closing this gap can provide invaluable insights into the nutritional implications of diets and inform the formulation of policy options to help the nation achieve its National Development Plan (NDP) goals and the UN SDGs (17).

In this study, we refer to both food security and food systems policies, while recognising that they address different aspects of the food landscape. Food security policy focuses on the dimensions of availability, access, utilisation, and stability. In contrast, food systems policy encompasses the broader processes, actors, and outcomes involved in producing, transforming, distributing, and consuming food, as well as the associated environmental and social impacts (4). Accordingly, we treat food security as one outcome within the broader food-systems agenda rather than as an equivalent or interchangeable concept.

South Africa implemented its initial comprehensive food security policy, the Integrated Food Security Strategy, in 2002. The primary goal of this strategy was to eliminate food insecurity, malnutrition, and hunger by 2015 (18). It aimed to achieve this by promoting household food production and trade, enhancing income generation, and creating more job opportunities. The policy also sought to improve nutrition and food safety, facilitate stakeholder dialogue, and strengthen their capacities (19). In 2010, South Africa introduced the New Growth Path (NGP) policy to boost growth, create employment opportunities, and promote equity (20). The NGP policy acknowledges food insecurity and refers to land, agricultural, and trade policies in this context (Table 1).

**Table 1:** South Africa’s food security and food systems policies.

Food security policy	Year implemented	Key objectives
Constitution of South Africa: Right to Food (Section 27)	1996	Establishes the right to sufficient food and water. Obligates the state to progressively realise this right.
Integrated Food Security Strategy (IFSS)	2002	Eliminate food insecurity and hunger. Promote household production and trade. Enhance income generation and job creation. Improve nutrition and food safety.
New Growth Path (NGP)	2010	Stimulate economic growth. Create employment and promote equity. Address food insecurity through the agricultural and trade sectors.
National Development Plan (NDP)	2012	Eliminate poverty and reduce inequality by 2030. Emphasise agriculture and rural development. Promote cross-sector collaboration.
National Policy on Food and Nutrition Security (NPFNS)	2013	Framework for national food and nutrition security. Integrate agriculture, health, trade, and social sectors.
Agricultural Policy Action Plan (APAP) 2015–2019	2014	Strengthen agricultural value chains. Support smallholder development. Enhance household food production.
National Food and Nutrition Security Plan (NFNSP) 2018–2023	2017	Improve food and nutrition security. Strengthen local food chains. Expand social protection and nutrition interventions.
Participation in UN Food Systems Summit	2021 ongoing	Align with global food-systems transformation. Focus on resilience, equity, nutrition, and small-scale farmers.
South Africa’s National Food and Nutrition Security Plan (NFNSP) 2025–2030	Ongoing	To enhance food security and reduce malnutrition in all its forms to ensure all South Africans live productive and healthy lives. Emphasize resilience through climate adaptation and digital innovation in agriculture. Build integrated multi-sectoral agri-food systems that promote equitable household food security, livelihoods, nutrition, and well-being.

In 2012, South Africa launched the National Development Plan (NDP) developed by the National Planning Commission (NPC) to eliminate poverty and reduce inequality by 2030 (21) (Table 1). One of the key focus areas of the NDP is agriculture, and it holds that access to food affects household food security (22). One of the critical features of the NDP is its advocacy for collaboration among government, the private sector, and civil society to establish local food systems that support food access and utilisation, as well as the broader food systems. The NDP “provides an innovative framework to begin to inform action required across society to deal with pervasive hunger” (23). The NDP advocates stakeholder engagement throughout the entire food systems, with numerous linkages across sectors and various government departments (15).

The most recent food security policy, the National Food and Nutrition Security Plan (NFNSP), was introduced in 2017 to enhance food security and nutrition. Led by the Office of the Deputy President and the Department of Planning, Monitoring and Evaluation (DPME) within the Presidency, it marked a significant departure from previous policies by involving multiple government departments and non-state actors (8). Key objectives included forming a multi-sectoral food and nutrition security council to coordinate policies and implementation, establishing inclusive local food chains to improve food access, expanding social protection and livelihood programs, and increasing efforts in nutrition interventions.

Currently, the South African government, led by the Department of Agriculture, Land Reform and Rural Development (DALRRD), is developing the new 2025–2030 National Food and Nutrition Security Plan (NFNSP) (Table 1). South Africa also actively participated in the United Nations Food Summit, reaffirming its commitment to global food security and sustainable development. The country engaged in discussions on agricultural resilience, nutrition, and equitable food systems, emphasising the need for international cooperation and support for small-scale farmers.

South Africa has had numerous food policies that primarily focus on food security, emphasising food production rather than the entire food system. This means that other aspects, such as the supply chain and the food environment, are often overlooked (10). Additionally, overarching food policies, such as the National Development Plan, the New Growth Path, and the Reconstruction and Development Programme, affect the food system (8). However, they fails to address the critical need for a comprehensive food security policy, although they do recognise the food production and consumption aspects of the food system (13). Therefore, South Africa needs a multi-dimensional strategy to transform its food system. This strategy should include prioritised policy options, which are interventions that are cost-effective, feasible to implement, and adaptable across a range of contexts while delivering benefits under different future conditions (24). In this study, prioritised policy options are defined as actions that generate meaningful benefits under a wide range of plausible future food-system conditions (25). These options are characterized by low implementation risk and a consistent contribution to improved nutrition, sustainability, and food-system resilience, regardless of how future uncertainties unfold.

The research gap lies not only in South African food policies, which have a limited focus on nutrition and often emphasise food security in terms of food quantity rather than diet quality, but also in the limited evidence on how food system policies can be designed and prioritised to improve the affordability of healthy diets. Existing research has largely examined food security, nutrition, or individual policy interventions in isolation, with less attention given to evaluating integrated food system policy options or identifying interventions that are feasible, cost-effective, and robust across different contexts. In addition, the fragmentation of food policies, with overlapping objectives, has led to incoherent implementation, yet there is limited evidence on how policy coherence can be strengthened to support healthier diets. Therefore, this study aims to explore and evaluate food system policy options, with a particular focus on prioritised strategies, to identify policy interventions that can improve the affordability of healthy diets and contribute to food system transformation in South Africa.

## 2 Materials and methods

### 2.1 Data sources

The study collected qualitative data through a systematic review using the PRISMA approach to identify publications on food systems that prescribe policy options to transform them. A systematic literature review, adapted from Hawkes et al. (26), was employed to extract policy options for transforming food systems from the relevant publications. Lastly, an online survey was distributed to stakeholders across the South African food system, who ranked the selected policy options according to their priorities. This process produced a final list of prioritised policies to transform the food system and ensure affordable, healthy diets.

### 2.2 Search strategy and eligibility criteria

A search strategy was developed to identify relevant publications using the keywords and Boolean operators listed in Table 2. Only publications written in English were considered, and those published from 2013 to 2023 were included, allowing the review to focus on the most recent decade of food systems research. The study used Scopus, PubMed, andWeb of Science, due to their broad scholarly coverage. In addition, a manual search was conducted on other web sources to access grey literature. Only publications with accessible full-text documents were included (10).

**Table 2:** Search strategy to identify food systems reports.

Keywords	Source databases			
Policy* OR strategy* AND transform* OR reform OR reorient* OR reshap* OR shift AND “food systems” AND afford* OR cheap* OR access* AND “health* diet*” OR “nutrient* diet*” OR “health* food” AND “South* Africa*” OR “SA”	Pubmed	Scopus	Web of Science	Other sources
	4	279	20	37
Filters				
Limit to English Language Limit to 2013–2023				

Source: Author’s computation (2023).

The next stage involved screening the titles and abstracts of the records by applying the inclusion and exclusion criteria. For records to be included in the analysis, they must meet the following criteria: the study must focus on holistically transforming food systems, enhance the affordability of healthy diets, provide policy recommendations to transform food systems to make healthy diets more affordable, and have been published between 2013 and 2023. South Africa was included as a search criterion in the systematic review. However, only one relevant article on food system policy in South Africa was identified within the selected publication period. As a result, the review necessarily drew on broader global literature, and the relevance of the identified policy options to the South African context was inferred and subsequently used for stakeholder prioritisation.

**Figure 1:**
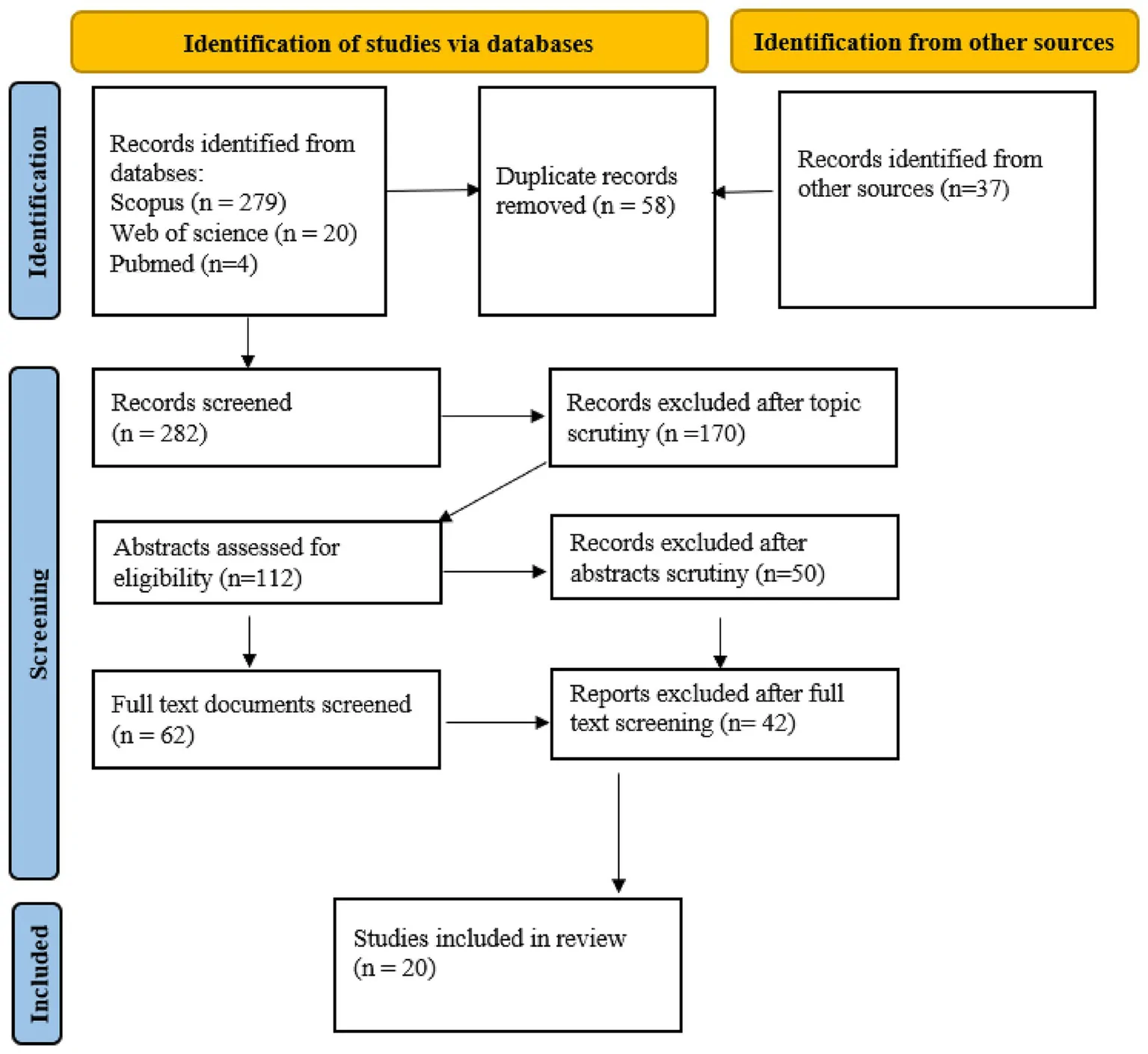
PRISMA flow diagram of the study. Source: Adapted from Page et al. (27), licensed under CC BY 4.0.

The PRISMA flow diagram, adapted from Page et al. (27), illustrates the identification of reports (*n* = 316) that provide policy recommendations for transforming food systems to achieve affordable, healthy diets from three databases and other sources. A total of 316 records were identified (279 from Scopus, 20 from Web of Science, 14 from PubMed, and 37 from other sources). After removing duplicates, 282 records were screened, of which 170 were excluded for lack of relevance to the topic. Of the 112 abstracts assessed, 50 were excluded for topic relevance, leaving 62 full-text documents for detailed review. Following full-text screening, a further 42 studies were excluded, leaving 20 studies for the final evaluation (Figure 1).

### 2.3 Identifying potential prioritised policy options

Following the PRISMA systematic review, the study adopted the four-step approach proposed by Hawkes et al. (28) to identify potential policy options. First, a systematic review was conducted to identify evidence-based expert reports containing clear policy recommendations for transforming food systems to support affordable, healthy diets. The screening process, guided by predetermined eligibility criteria, yielded 20 reports that met the inclusion criteria (Table 3). Second, relevant policy options were extracted from the selected reports. Third, policy options with similar intended impacts were consolidated and merged. Finally, the resulting recommendations were refined and classified according to their pathways to impact.

**Table 3:** Details of the 20 documents included in the systematic review.

No.	Paper included	Title of the paper/report
1	(2)	Reverse thinking: taking a healthy diet perspective toward food systems transformations
2	(4)	Food security and nutrition: building a global narrative toward 2030
3	(5)	Nutrition-sensitive agriculture and food systems in practice. Options for intervention
4	(17)	Changing diets and the transformation of the global food system
5	(19)	*20*20 Global nutrition report: Action on equity to end malnutrition
6	(37)	Policy options for food systems transformation in Africa - from the perspective of African universities and think tanks
7	(54)	Toward food systems transformation—five paradigm shifts for healthy, inclusive and sustainable food systems
8	(38)	Food in the Anthropocene: the EAT–Lancet Commission on healthy diets from sustainable food systems.
9	(33)	A diagnostic framework for food system governance arrangements: The case of South Africa. NJAS-Wageningen Journal of Life Sciences.
10	(34)	Food and nutrition policy in South Africa: the national vision, policy space, and policy alignment (Doctoral dissertation, Stellenbosch: Stellenbosch University).
11	(42)	Sustainable and nutrition-sensitive food systems for healthy diets and prevention of malnutrition in Europe and Central Asia. Food and Agriculture Org.
12	(39)	Food systems and diets: Facing the challenges of the 21st century
13	(68)	Transforming food systems: The missing pieces needed to make them work
14	(35)	Achieving food system transformation: Insights from a retrospective review of nutrition policy (in)action in high-income countries
15	(36)	Connecting the dots: Policy innovations for food systems transformation in Africa
16	(63)	Transforming food systems to achieve healthy diets for all
17	(43)	Policy considerations for African food systems: toward the United Nations 2021 Food Systems Summit. Sustainability
18	(44)	Africa regional overview of food security and nutrition 2020: Transforming food systems for affordable healthy diets (Vol. 1). Food & Agriculture Org.
19	(61)	Science and innovations for food systems transformation
20	(51)	Searching for the essential: Exploring practitioners’ views on actions for re-orienting food systems toward healthy diets.

Source: Author’s computation.

### 2.4 Steps to prioritise policy options to transform food systems

To prioritize South Africa’s policy options identified through the systematic review, this study conducted a stakeholder survey using key informant interviews. Purposive sampling was used to select 62 stakeholders with expertise and experience across the South African food system. To ensure geographic representation and capture the diversity of food-system contexts across the country, participants were selected from all nine provinces. The sample included stakeholders from key food-system sectors, including the private sector (e.g., farmers, food producers, retailers, and agribusinesses), academia (universities and research organizations), non-governmental and development organizations, the public sector (health, agriculture, trade, and development), and civil society organizations involved in food consumption, food security, and advocacy. The participant composition comprised 20 representatives from the private sector, 14 from development organizations, 11 from the public sector, 9 from civil society, and 8 from academia. This sampling approach enabled the researcher to engage individuals with diverse perspectives and specialized knowledge of the South African food system, while also accounting for their availability and willingness to participate.

The participants were selected based on their knowledge and experience in food systems research on healthy diets, as well as their availability and willingness to participate in the study. A Google Form was developed that contains policy options identified in the systematic review and was grouped into seven themes derived from the PRISMA review. The form outlined the study objectives and explained how participants’ responses would be used. Participants were asked to select the most and least important policy options for transforming food systems to support affordable, healthy diets in South Africa, to suggest any missing options, and to provide justifications for their choices.

Ethical approval for this study was granted by the University of Pretoria’s Ethics Committee on 20 July 2023 (Reference: NAS311/2022). All participants gave written informed consent before completing the survey.

### 2.5 Data analysis

Thematic analysis was used to analyse qualitative data from reports identified through the PRISMA systematic review and from the online survey to prioritise policy options. Thematic analysis is a qualitative method used to identify and interpret patterns of meaning across textual data. In this study, an inductive approach to thematic analysis was applied to systematically code the extracted data and develop data-driven themes capturing key concepts across the included documents (29). ATLAS.ti software was used to support report coding. During the first phase, line-by-line coding was conducted to identify and categorise policy options through close examination of the text.

The Best-Worst Scaling method was used to rank each policy option as either ‘best’ or ‘worst’ after participants completed the survey. An aggregate BW score was calculated by subtracting the aggregate ‘least’ value from the aggregate ‘most’ value for each policy option (30). In the survey, participants’ most- and least-important votes were counted from a total of 62. To validate the BWS results, we examined several aspects: internal consistency, model reliability, convergence, and robustness. A limitation of the systematic review in this study is that it was conducted without a team-based approach, which may increase the risk of bias.

## 3 Results and discussion

### 3.1 Geographical focus of the food systems transformation reports

Regarding the geographical focus of the studies, 70% of the reviewed documents had a global scope. These studies provided policy options for promoting healthy diets that were not specific to any particular country or region. Only 30% of the studies focused on Africa, and just one global study identified policy options relevant to South Africa at the time of the database search. This suggests that food system policy has received limited research attention in South Africa. On the other hand, most of the papers (85%) were published after 2020, reflecting the growing global interest in food systems transformation.

### 3.2 The thematic focus of the studies

The thematic analysis identified seven broad themes across the 20 documents included in this review, with all themes represented in the full set of papers. Governance policy options were the most dominant theme, accounting for 19% of the representation, followed by agricultural, research, technology and innovation, with 16%. Financial, supply chain, and regulatory policy options were tied at 13%, while education and awareness policy options were the least mentioned in the reports. The systematic review identified policy options crossing multiple thematic areas, reflecting the broad scope of food system interventions reported in the literature. According to (31), transforming food systems requires a holistic approach because they are characterised by complex interactions among production, food environments, consumer behaviour, health, and environmental sustainability. Furthermore UNDP (32), emphasises the importance of strong policy coherence to ensure that policies across sectors are aligned toward a common objective and implemented in a coordinated manner.

Governance policy options were identified in four out of 20 documents (33–36). National food-based dietary guidelines (FBDGs) were mentioned in three out of the 20 included documents (17, 37, 38). Regulatory policy options were identified in three out of 20 documents (36, 38, 39). Most of these policy options (50%) mentioned restricting the marketing and advertising of unhealthy food, especially to children.

Financial policy options to address the affordability of healthy diets, such as taxes on unhealthy foods and beverages, subsidies and food and cash vouchers to improve the purchasing power and diet quality of vulnerable groups, were included in three out of 20 policy documents. Nine documents highlighted the importance of raising awareness to equip consumers with nutritional knowledge. Among these, two specific actions were outlined, including the use of mass media and digital interventions to influence consumer behaviour, promote healthier diets, and encourage active lifestyles. Moreover, one study identified the need for complementary interventions, such as counselling, to support app-based interventions.

Policy options related to the supply chain were identified in 12 of 20 documents, with actions to build agricultural and market infrastructure, such as roads, transportation, postharvest handling, and storage, mentioned in eight of them. Food safety training for food producers, as well as supporting access to farmer markets and e-commerce platforms, was critically considered to ensure safe, nutritious food in markets.

Agricultural policy options were identified in eight out of 20 documents. All documents recommended increasing the availability of healthy food by sustainably diversifying crops and promoting the production and consumption of nutritious indigenous crops. The strategies for diversifying agricultural production included providing incentives, subsidising farmers who produce healthy food, and developing climate-smart agriculture. Women’s empowerment, achieved through control and ownership of productive resources and the provision of training and agricultural assets, was the second most frequently mentioned recommendation.

Research and innovation policy options were identified in eight out of 20 documents. The primary focus of this thematic area was on food and biofortification to enhance the nutrient profile of affordable foods widely consumed by households facing financial constraints. According to (40), the introduction of food fortification in South Africa in 2003 exemplifies the food system paradox, in which appropriate policy responses have not yielded the expected results. However Barkhuysen (41), highlights an improvement in the country’s vitamin A status, which could be attributed to the National Food Fortification Programme and vitamin A supplementation programmes.

### 3.3 List of policy options extracted from the systematic review

Following the identification of eligible reports for inclusion in the study, 31 policy options were extracted using the systematic review methodological framework proposed by (28). These options were subsequently refined into “no-regrets” policy options through stakeholder engagement processes. The policy options were derived from a systematic review of food systems reports, which provided detailed recommendations for transforming food systems toward healthier diets. All recommended actions identified across the reports were systematically extracted, documented in detail, and subsequently filtered and consolidated through a structured mapping of pathways to impact. This process examined how each policy option could improve the availability, accessibility, and affordability of healthy food. Table 4 summarises the potential contribution of each policy option to food system transformation in South Africa.

**Table 4:** Extracted policy options from the reviewed reports.

No	Themes identified through PRISMA	Impact of the policy option
Agricultural options		
1	Deliver urban and peri-urban agricultural programmes.	Increase the availability of fresh, nutritious foods in proximity markets and the access of urban residents to a diversified, nutritious diet.
2	Provide agricultural extension programmes to train farmers to produce nutritious foods.	Diversification approaches increase the availability and affordability of diverse foods. Increase the production and affordability of healthy food to the local population
3	Promote production and consumption of indigenous foods	
4	Provide for women and the vulnerable households with agricultural assets and training	
5	Redesign agricultural development programmes to focus on nutritious food	
Financial policy options		
6	Subsidise healthy foods	Subsidies for selected nutritious foods can increase the affordability of healthy diets and/or incentivize purchase
7	Tax on unhealthy foods and beverages	Taxes on ultra-processed foods (e.g., sugar-sweetened drinks) can also be used to increase prices and restrict consumption.
8	Provide cash or vouchers to vulnerable groups	Social protection helps families to increase their consumption and to access more and better food while also helping to develop their productive asset base, which is critical for sustaining good nutrition in the long term and facilitating access to health care and services.
9	Provide financial and technical support to small businesses	Increases the availability and affordability of healthy foods
Regulatory policy options		
10	Restrict advertisements and marketing of unhealthy foods	Reduces the exposure of children to unhealthy foods, especially snacks and limits consumers’ chances of buying unhealthy foods
11	Mandatory limits on certain nutrients	Reduces the availability of unhealthy nutrients such as sugars/salts/fats in foods available to local populations
12	Mandatory nutrition labelling on food packages	Informs consumers about foods with healthier nutrition profiles and can also motivate manufacturers to produce foods with healthier nutrition profiles.
13	Zoning laws to restrict the sale of unhealthy foods	Reduce the availability of unhealthy foods, especially in areas like schools
Supply chain policy options		
14	Training programmes on storage, processing and packaging to ensure food safety	Improves the accessibility of nutritious and safe foods in ways that address modern food environments
15	Establish short food supply chains	Prevents and reduces food loss, especially through short value chains
16	Improve infrastructure in agriculture and market systems	Improve the availability in markets and the affordability of nutritious foods
17	Facilitate access to food markets for smallholder farmers and SMMEs	Increases the availability of nutritious foods in markets serving local populations
18	Reduce loss and waste of nutritious foods	Ensures the availability of healthy food to the local populations
Governance policy options		
19	Ensure coherence and collaboration between different ministries	Improves food quality, food safety and food system sustainability for broad categories of stakeholders
20	Demystify the policy development process	Strengthens evidence-based solutions as far as food systems’ ability to deliver nutritious foods is concerned
21	Adopt a public food procurement policy that applies nutritional guidelines	Increases availability, affordability and access of nutritious foods and reduces access to foods high in fats, sugars and salt to people served by public institutions
22	Trade policies that prioritise healthy foods	increases the availability of nutritious foods in importing countries, especially in counter-seasonal periods
23	Develop Food-based dietary guidelines	Increases the availability, affordability and appeal of nutritious foods, and reduces the availability, affordability and appeal of foods high in fat, sugar and salt to all populations
Awareness and education policy options		
24	advocacy on healthy foods and raising awareness via mass media	Effective nutrition education ensures that increased food production/income translates into improved diets and improved nutrition status
25	Providing nutrition education and counselling to women	May improve some maternal and infant health and behavioural nutrition outcomes
26	Make healthy foods default options on menus of restaurants	Nudges patrons to make healthy food choices in food establishments
Research and innovation policy options		
27	Reformulation of processed foods	Reduces the availability of fats, sugars and salt in foods already available to all populations
28	Implement biofortification programmes	Increases the availability of micronutrients in staple foods already available to consumers
29	Implement food fortification programmes	Substitutes the consumption of nutrient-poor varieties with nutrient-rich ones
30	development of processed products that extend the shelf life of healthy food	Increases the availability, affordability and appeal of nutritious foods throughout the year to all populations
31	Develop alternative sources of protein	Increases the availability, affordability and appeal of alternative micronutrient-rich protein sources and reduces the appeal of red meat to high red-meat consumers

Source: Author’s computation.

### 3.4 Prioritisation of policy options

**Figure 2:**
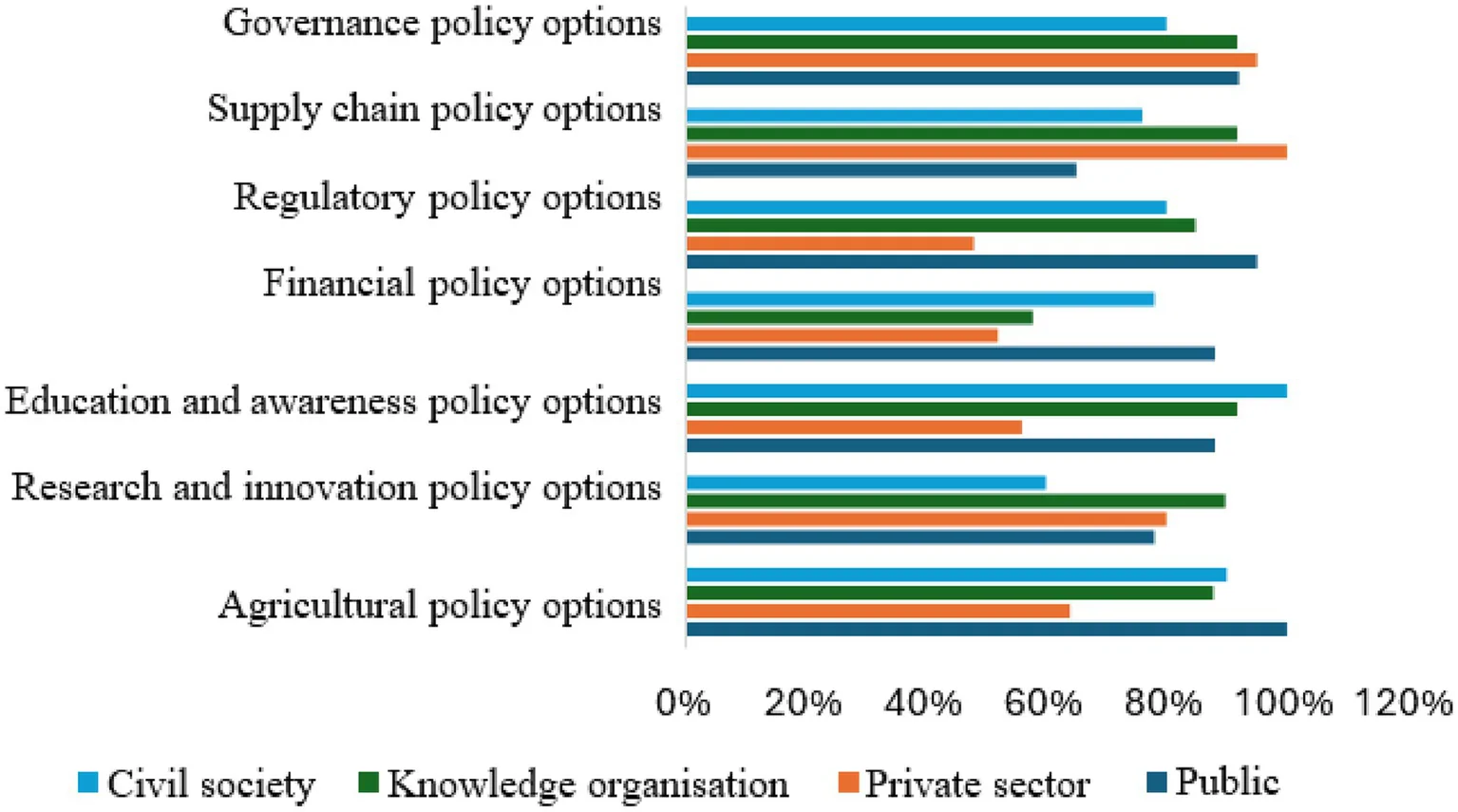
Prioritisation of themes by participant’s categories.

After the systematic literature review and extraction of the policy options, 62 participants were asked to rank each of the 31-policy options under the different themes as ‘most’ or ‘least’ important when it comes to transforming South Africa’s food system toward affordable healthy diets. The votes were scored out of 62, the total number of respondents who completed the survey.
The results presented in Figure 2 indicate clear variation in stakeholder preferences regarding food system transformation policies in South Africa. Overall, civil society actors show the strongest preference for education and awareness policies, suggesting a focus on behavioural change and public engagement as key levers for transformation. Knowledge organisations consistently prioritise research and innovation policies, reflecting an emphasis on generating evidence and advancing technology. The private sector demonstrates relatively greater support for governance and regulatory policy options, indicating interest in institutional and rule-based frameworks that shape market operations, and shows moderate support for supply chain and financial policies. In contrast, public sector respondents tend to prioritise agricultural and supply chain policy options, highlighting a production- and system-efficiency-oriented perspective. Across all groups, no single policy category dominates, underscoring divergent priorities among stakeholders. These differences reflect varying institutional roles, mandates, and interpretations of the most effective entry points for food system transformation, underscoring the importance of inclusive, multi-stakeholder approaches in policy design.

### 3.5 Ranking policy options

Thirty-one potential policy options identified through the systematic review were presented to participants via a Google Form survey to determine which were considered “most” and “least” important for transforming South Africa’s food system toward affordable and healthy diets. Following the calculation of the Best–Worst (BW) scores for each policy option, 20 out of the 31 policy options were retained for further analysis. On average, policy options classified as most important accounted for 78% of responses, whereas those classified as least important accounted for 22% (Table 5). These findings highlight stakeholder priorities regarding food system transformation in South Africa and reflect growing policy interest in improving food affordability, nutritional outcomes, and the sustainability of the national food system.

**Table 5:** Ranking of policy options by vote percentage and BW scores.

Policy options	Most important votes	Least important votes	BW score
Introduce mandatory nutrition labelling on food packages to reflect nutrient composition	96%	4%	0.92
Improve infrastructure in agriculture and market systems to increase the availability of nutrient-dense foods	96%	4%	0.92
Demystify the policy development process	96%	4%	0.92
Advocacy on healthy foods and raising awareness via mass media regarding processed food	96%	4%	0.92
Provide agricultural extension programmes to train farmers to produce nutritious foods	91%	9%	0.82
Ensure coherence and collaboration between ministries that are affected by food security and nutrition.	91%	9%	0.82
Provide women and vulnerable households with training, education and resources to enhance agricultural productivity and incomes.	89%	11%	0.78
Impose mandatory limits on sugar, salt and fats in packaged foods	87%	13%	0.74
Develop infrastructure to reduce the loss and waste of nutritious foods and increase its redistribution	87%	13%	0.74
Deliver urban peri-urban agricultural programmes	85%	15%	0.70
Facilitate access to food markets for smallholder farmers and SMMEs by establishing cooperatives, hubs and developing mechanisms for collective bargaining and increasing access to price information	85%	15%	0.70
Promote the production and consumption of indigenous foods	81%	19%	0.62
Restrict advertisements and marketing of unhealthy foods	81%	19%	0.62
Develop Food-based dietary guidelines	81%	19%	0.62
Providing nutrition education and counselling to women	81%	19%	0.62
Establish or support short food supply chains	79%	21%	0.58
Trade policies that prioritise the export of healthy foods and reduce barriers to their import	79%	21%	0.58
Reformulation of processed foods to reduce the levels of sugar and fats	79%	21%	0.58
Redesign agricultural development programmes to focus on producing nutritious food	77%	23%	0.54
Implement food fortification programmes	77%	23%	0.54
Reduce the prices of healthy diets by subsidising healthy foods	77%	23%	0.54
Lower the price of healthy foods by providing financial and technical support to small businesses that sell healthy food	74%	26%	0.48
Adopt a public food procurement policy that applies nutritional guidelines to food procured for public institutions such as schools and prioritise purchasing from smallholder farmers	74%	26%	0.48
Use zoning laws to restrict the sale of unhealthy foods in specific areas such as schools and clinics	72%	28%	0.44
Provide training programmes for food producers and retailers on storage, processing and packaging to reduce spoilage and contamination of nutritious foods	72%	28%	0.44
Provide social protection in the form of cash or vouchers to enable vulnerable groups to afford healthy diets	70%	30%	0.40
Reformulation of processed foods to reduce the levels of sugar and fats	68%	32%	0.36
Impose taxes on unhealthy foods and beverages to discourage consumers from purchasing such foods/beverages.	66%	34%	0.32
Implement biofortification programmes	62%	38%	0.24
Development of processed products that extend the shelf life of healthy food	61%	39%	0.22
Develop alternative sources of protein	57%	43%	0.14
Make healthy foods default options on menus of restaurants	30%	70%	−0.40

Source: Author’s computation.

### 3.6 Prioritised policy options

Table 6 presents the final prioritised list of the final 20 policy options to transform South Africa’s food system for affordable, healthy diets. These policy options were prioritised based on their BW scores; i.e., only those with a BW score of over 0.5 were included in the final list. The Best–Worst Scaling results show strong internal consistency, logical coherence, and a clear differentiation among policy priorities. Therefore, the findings of BWS should be considered reliable and valid representations of stakeholders’ priorities for improving food and nutrition policies. For instance, The Best–Worst Scaling results showed strong internal consistency, with high “most important” votes aligning with low “least important” votes and consistently high BW scores. The smooth decline from 0.92 to 0.54 scores, with no erratic jumps or reversals, indicates good sensitivity and confirms that the rankings are stable and robust to minor variations. The policy options are described as follows.

**Table 6:** List of prioritised policy options to transform South Africa’s food systems.

Policy options	Most important votes	Least important votes	BW Score
Introduce mandatory nutrition labelling on food packages to reflect nutrient composition	96%	4%	0.92
Improve infrastructure in agriculture and market systems to increase the availability of nutrient-dense foods	96%	4%	0.92
Demystify the policy development process	96%	4%	0.92
Advocacy on healthy foods and raising awareness via mass media regarding processed food	96%	4%	0.92
Provide agricultural extension programmes to train farmers to produce nutritious foods	91%	9%	0.82
Ensure coherence and collaboration between ministries that are affected by food security and nutrition.	91%	9%	0.82
Provide women and vulnerable households with training, education and resources to enhance agricultural productivity and incomes.	89%	11%	0.78
Impose mandatory limits on sugar, salt and fats in packaged foods	87%	13%	0.74
Develop infrastructure to reduce the loss and waste of nutritious foods and increase its redistribution	87%	13%	0.74
Deliver urban peri-urban agricultural programmes	85%	15%	0.70
Facilitate access to food markets for smallholder farmers and SMMEs by establishing cooperatives, hubs and developing mechanisms for collective bargaining and increasing access to price information	85%	15%	0.70
Promote the production and consumption of indigenous foods	81%	19%	0.62
Restrict advertisements and marketing of unhealthy foods	81%	19%	0.62
Develop Food-based dietary guidelines	81%	19%	0.62
Providing nutrition education and counselling to women	81%	19%	0.62
Establish or support short food supply chains	79%	21%	0.58
Trade policies that prioritise the export of healthy foods and reduce barriers to their import	79%	21%	0.58
Redesign agricultural development programmes to focus on producing nutritious food	77%	23%	0.54
Reduce the prices of healthy diets by subsidising healthy foods	77%	23%	0.54
Implement food fortification programmes	77%	23%	0.54

Source: Author’s computation.

#### 3.6.1 Agricultural policy options

Twenty-five percent of policy options under the agricultural policy sector were prioritised and included in the final list of prioritised policy options to transform food systems (5, 37, 42–44). The most-voted policy option in this theme is the provision of agricultural extension programmes to train farmers to produce nutritious foods. The necessity of this policy option is supported by Hendriks et al. (45), who recommended changes in producer support programmes and extension practices. These changes aim to broaden the focus beyond producing staple crops to promoting and supporting agricultural systems that increase the production of nutritious foods. Misselhorn and Hendriks (46) further indicated that extension services can be compounded by access to resources such as market information, credit facilities, infrastructure provision and the removal of barriers to market entry.

The second policy option under this policy sector is the provision of training and agricultural resources to women and vulnerable households, which is critical in reducing the barriers women face in controlling productive assets and increasing productivity (46). The third policy option under this policy sector was to deliver urban and peri-urban agricultural programmes. Urban and peri-urban agriculture is a crucial policy option with the potential to improve dietary diversity in urban areas with limited nutritious food options. Yet in most cities, there is little government support for urban and peri-urban agriculture and instead restrictive regulations that limit local food production, such as rising land values (47).

The fourth policy option under this policy sector was the promotion of the production and consumption of indigenous foods. This finding is corroborated by (48), who argues that most of South Africa’s health, food, and agricultural policies focus on general food production and consumption rather than access to fresh fruit vegetables and indigenous foods. Therefore, it is recommended that food and nutrition policies be aligned to improve the availability, affordability, and consumer preference for indigenous crops and other fresh produce, particularly because these foods are often more affordable than alternative food options (49).

The fifth policy option under this policy sector was the redesign of agricultural development programmes to focus on producing nutritious food Igumbor et al. (50). argues that this policy option is necessary, as it has the potential to increase the availability of nutritious foods, especially by diverting agricultural support to incentivise the production of more diverse, nutrient-rich crops rather than staples that tend to be less nutritious but high in energy.

#### 3.6.2 Governance policy options

Twenty percent of policy options under the governance policy sector were prioritised and included in the list of prioritised policy options (33–36, 51). The first policy option was to demystify the policy development process (Table 6). The policy development process consists of a sequence of different stages, including problem identification, agenda setting, consideration of policy options, decision-making, implementation, and monitoring and evaluation. Therefore, this stage-based view requires the involvement of all government actors to strengthen evidence-based solutions for food systems (52, 53). The merits of moving toward improved stakeholder engagement include potentially responsive policies to the needs of diverse actors, particularly those marginalised within the food system (22). It also has the potential to strengthen the problem identification process, resulting in well-thought-out, coherent, and generally acceptable solutions.

The second policy option was to ensure coherence and collaboration between different ministries that deal with or are affected by food security and nutrition. Coherent policies are necessary to promote more inclusive and nutrient-sensitive investment, thereby enhancing food quality, food safety, and food system sustainability for various stakeholders. Both market incentives and public regulation are necessary to support connectivity and enhance responsiveness (54). Furthermore Thow et al. (55), argues that policy incoherence arises from tensions between competing policy objectives and a lack of coordination across government departments. In South Africa, this is evident where economic policies, such as those relating to trade and agriculture, may prioritise economic growth, employment, and food production, while nutrition and health policies focus on improving diet quality and reducing diet-related non-communicable diseases (55).

As a result, food system policies are often fragmented, with overlapping mandates and government departments operating in silos, leading to duplication of activities and inconsistent implementation (55). Although greater multisectoral collaboration is widely recognised as a key policy option for food system transformation, achieving it in practice is challenging due to differing institutional mandates, competing priorities, and resource constraints. Addressing these barriers will require stronger governance mechanisms, clearly defined roles and responsibilities, coordinated policy planning, and accountability structures that align the objectives of different sectors toward improving food security and healthy diets.

The third policy option was to develop Food-based Dietary Guidelines, for which South Africa currently has prescribed FBDGs but still lacks data on nationally consumed foods and on indigenous diets (55). The fourth policy option was to align trade policies with nutrition objectives by promoting access to healthy foods while reducing barriers to their import where appropriate. This supports the global view that trade policy should form part of a broader strategy to improve healthy diets. However, the nutritional impacts of trade policies depend on how they are designed and implemented. In South Africa, export-oriented production of high-value fruits and vegetables can generate economic benefits and support agricultural growth, but it does not necessarily improve domestic access to these foods and may coincide with increased imports of inexpensive, highly processed foods. Consequently, trade policies should be complemented by domestic measures, including investments in local food production, support for smallholder farmers, and social protection programmes, to ensure that nutritious foods remain available and affordable for the domestic population while protecting vulnerable households from food price volatility (56).

#### 3.6.3 Supply chain policy options

Twenty percent of policy options under the supply chain policy sector were prioritised for inclusion in the prioritised policy options list (5, 36, 39, 44). The most-voted policy option on this list was improving agricultural and market infrastructure to increase the availability of nutrient-dense foods. Nutrient-dense foods tend to be more perishable; therefore, improving postharvest handling is crucial for enhancing their availability and affordability (57). The second policy option under this theme was to develop infrastructure to reduce food loss and waste and increase their redistribution. According to Olson et al. (52), food loss and waste are prevalent across both the production and consumption cycles in South Africa (Table 6).

Furthermore, the 2015–2019 Agricultural Policy Action Plan identifies that small-scale farmers often lack access to agricultural technologies. This challenge, combined with the poor rural infrastructure and low adoption of new technology across the food system, including postharvest and food processing technologies, contributes to significant postharvest losses. These factors also act as a major barrier to market access for many smallholder farmers in South Africa. Factoring in the current and future role of technological innovations in the food system, particularly in agricultural production, is crucial for developing and implementing effective national food security and nutrition policies and plans (21).

The third policy option was to facilitate access to food markets for smallholder farmers and small, medium, and micro enterprises (SMMEs) by establishing cooperatives and hubs, and by developing mechanisms for collective bargaining and increasing access to price information. Not only is this policy necessary to help South African smallholder farmers who struggle with market entry, but, according to Olson et al. (52), it can also increase the availability of nutritious foods in markets serving local populations. The fourth policy option was to establish or support short food supply chains. Short food supply chains are systems in which the number of intermediaries between producers and consumers is minimised, typically involving shorter physical distances and more direct relationships.

#### 3.6.4 Regulatory policy options

Fifteen percent of policy options under the regulatory theme were prioritised and included in the list of prioritised policy options in Table 4 (36, 38, 39). The most voted policy option was the introduction of mandatory nutrition labelling on food packages to reflect the food’s nutrient composition (Table 6). Food labelling in South Africa is already mandatory and is regulated under R146 of the Foodstuffs, Cosmetics and Disinfectants Act of 1972, which came into effect in 2012 (58). Currently, nutrition information is primarily displayed on the back of food packages, requiring consumers to interpret detailed nutrient information that can be difficult to understand.

According to Taillie (59), South Africa should consider implementing front-of-pack labelling (FOPL), such as warning labels indicating products high in sugar, sodium or saturated fat, to help consumers identify less healthy food products and make more informed choices. Evidence from international studies indicates that interpretive FOPL can improve consumers’ understanding of product nutritional quality and influence purchasing intentions, while FOPL policies may also encourage manufacturers to reformulate products. Evidence from a randomised controlled trial conducted among South African households found that warning labels improved the identification of products high in nutrients of concern and reduced reported intentions to purchase unhealthy products compared with other labelling formats (58). However, the effectiveness of FOPL depends on appropriate policy design, consumer understanding, regulatory implementation and monitoring. In the South African context, FOPL should therefore build on the existing food-labelling regulatory framework while taking into account local socioeconomic and food-system conditions.

The second policy option was to impose mandatory limits on sugar, salt and fats in packaged foods. The relevance of this policy option for South Africa is supported by Teagle (48), who found that countries that have pioneered such restrictions have seen dramatic reductions in food fat content and related diseases, particularly among the most vulnerable socioeconomic groups that cannot afford healthier food options. The third policy option is to strengthen restrictions on the advertising and marketing of unhealthy foods. According to Herforth (60), stronger enforcement of regulations targeting private-sector practices, particularly the marketing of unhealthy foods and beverages, breastmilk substitutes, and food advertisements directed at children, is essential to reduce consumption of unhealthy products. In the South African context, this could involve strengthening existing regulations on food marketing to children across television, digital media, and other advertising platforms, as well as improving monitoring and enforcement mechanisms to ensure compliance. Given the increasing exposure of children to digital marketing and the high burden of diet-related non-communicable diseases in South Africa, restricting the promotion of unhealthy foods could support healthier food choices and complement other nutrition policies, such as front-of-pack food labelling and nutrition education.

#### 3.6.5 Research and innovation policy options

Ten percent of policy options under the research and innovation were included in the final list of prioritised policy options (36, 61). Implementing food fortification programmes is the only policy option under the research and innovation policy sector included in the final list of prioritised policy options in Table 6. Olson et al. (52) state that food fortification is a proven, safe, and cost-effective strategy for improving diets and preventing and controlling micronutrient deficiencies. In 2003, South Africa made the fortification, with vitamin A, thiamine, riboflavin, niacin, pyridoxine, folic acid, iron and zinc of certain types of maize meal and wheat flour is mandatory following the promulgation of R504 Regulations relating to the fortification of certain foodstuffs. Additionally Keats et al. (62), state that there is strong evidence that food fortification in high-income countries is effective in addressing micronutrient deficiencies; however, evidence remains lacking in low- and middle-income countries.

#### 3.6.6 Awareness and education policy options

Two policy options (19, 63) (10%) under the awareness and education theme were prioritised for inclusion in the policy options list in Table 6. The first policy option is to advocate for healthy foods and raise awareness about processed foods through mass media. One of the six strategic objectives of the National Food and Nutrition Security Plan (2018–2023) is to encourage people to make informed food and nutrition choices through an integrated communication strategy (34). With South Africa having a high obesity rate, with about half of all adults being either overweight or obese, mass media campaigns that raise awareness about healthy eating are critical to helping consumers make informed food choices. South Africa’s food environment is characterised by aggressive advertisements promoting unhealthy foods and beverages, making it challenging for consumers to adopt healthy diets (64).

In addition, these campaigns should target low-income communities that consume high amounts of energy-dense, nutrient-poor foods. In these communities, a lack of education and low income levels prevail, and increased awareness through mass media can improve knowledge and help the government reduce unhealthy food consumption (65). The second policy option was to provide nutrition education and counselling to women, particularly during pregnancy. Thow et al. (55) recommends coupling this intervention with the consistent use of micronutrient supplements, food supplements, or fortified foods. However, nutrition education alone is unlikely to improve dietary practices where poverty and the high cost of healthy foods limit food choices. In the South African context, nutrition education should therefore be complemented by interventions that improve the affordability and availability of nutritious foods.

#### 3.6.7 Financial policy options

Five percent of the financial policy options were included in the final list of prioritised policy options in Table 6 (43). The only policy option under the financial theme is subsidising healthy foods to reduce prices. According to (66), interventions that promote healthful behaviours, such as subsidies, generally have larger effect sizes than those that target the cessation of unhealthful behaviours. According to (67), subsidies have a greater positive effect in traditional food systems, which are characterised by smallholder production and shorter local food supply chains. In these systems, subsidies can lower food prices while supporting local producers, increasing both the availability and affordability of nutritious foods. Furthermore, subsidies need to be closely tailored to each food system context, as evidence of their effectiveness varies notably across contexts (32). Given South Africa’s dualistic food system, subsidies can reduce the prices of healthy foods by lowering the cost of inputs for smallholder farmers. Furthermore, targeting low-income groups, especially rural households, can reduce unequal access to nutritious foods (40).

**Figure 3:**
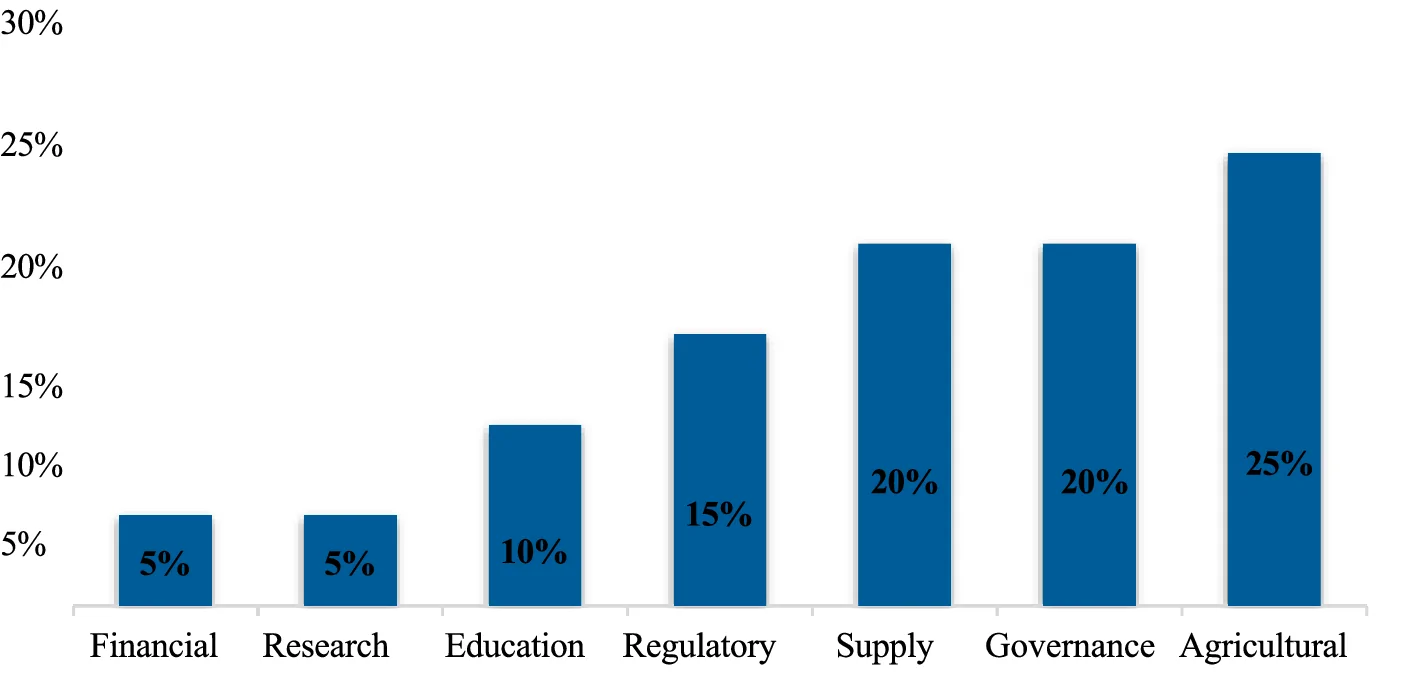
Priority policy options by themes. Source: Author’s analysis.

In summary, based on the final list of 20 prioritised policy options presented in Table 5, agricultural policy options are the most dominant, while financial and research and innovation policies are the least represented. Figure 3 summarises the South African food system policy options and indicates that they are primarily focused on agricultural interventions (25%), followed by supply-chain improvements (20%) and governance measures (20%). This highlights the importance of food production, distribution efficiency, and effective institutional coordination in achieving food security. Regulatory policies account for 15%, reflecting the role of standards, trade, and environmental regulations, while educational initiatives contribute 10% through capacity building, extension services, and nutrition awareness. Financial support and research interventions each account for 5%, indicating a comparatively low emphasis despite their importance in enabling innovation, investment, and long-term food system resilience. Overall, the findings show that policy priorities are concentrated on practical and structural interventions that directly influence food availability, access, and overall system performance.

### 3.7 Implementation feasibility and contextual considerations

The prioritisation of policy options reflects stakeholders’ views on their importance and feasibility, but does not guarantee their execution without obstacles in the South African context. The focus on agriculture, supply chains, and governance indicates that stakeholders considered interventions targeting food production, distribution, and institutional coordination to be especially important for enhancing the affordability of healthy diets in South Africa. However, the implementation will depend on affordability, cost-efficiency, institutional capacity, available resources, and intersectoral coordination, as well as the nation’s current food system and socioeconomic status.

The feasibility and effectiveness of these policy options may vary across South Africa, given differences in food-system frameworks, infrastructure, markets, and institutional capabilities. Evidence from other countries can guide policy formulation, but should not be considered directly applicable to South Africa. Consequently, prioritised policy options should be adapted to local contexts and, where appropriate, supported by monitoring and evaluation to assess their effectiveness and inform implementation. This underscores the need for a coordinated and complementary approach that addresses South Africa’s specific food-system challenges rather than relying on individual policy measures.

A limitation of this study was that the systematic review was conducted by a single researcher, which may have increased the risk of selection and interpretation bias. Only English-language publications from 2013 to 2023 were included, potentially excluding relevant studies in other languages and earlier evidence. Searches were conducted in Scopus, PubMed, and Web of Science, supplemented by manual searches of grey literature. However, only studies with accessible full-text documents were included, potentially excluding relevant evidence.

## 4 Conclusion and policy recommendations

The findings indicate that no single policy option is likely to be sufficient to improve the affordability of nutritious diets in South Africa. Rather, a combination of complementary interventions across the food system is needed. The identification of prioritised policy options across agriculture, finance, governance, regulation, supply chain, education and awareness, and research and innovation confirms the breadth of systemic challenges. While agriculture, governance, and supply chain interventions have emerged as the most emphasised areas, the relative neglect of research and innovation reveals a significant vulnerability in the long-term sustainability of the food system. The broad agreement among stakeholders on the prioritisation of policy options reinforces the importance of coordinated, complementary interventions to improve the affordability of nutritious diets in South Africa. Therefore, there is no single policy solution; instead, a coordinated, inclusive, and cross-sectoral approach will be necessary to achieve lasting change.

This study contributes to the evidence base by identifying and prioritising food system policy options that can improve the affordability of nutritious diets in South Africa. By combining evidence from the literature with stakeholder prioritisation, the study identified a set of prioritised policy options that are considered both important and feasible within the South African context. The findings highlight the need to strengthen policy coherence across sectors, improve access to affordable, nutritious foods, and integrate nutrition objectives into food system policies. These findings provide policymakers with an evidence-informed framework for prioritising interventions that support healthier diets and the transformation of the food system.

## Data Availability

The datasets used and/or analysed during the current study are available from the corresponding author upon reasonable request. All data generated or analysed during this study are included in this published article.
